# Digital selfie editing shows sex specific associations between processing biases and life satisfaction

**DOI:** 10.1038/s41598-025-99056-y

**Published:** 2025-04-24

**Authors:** Kalai Hung, Michella Feldborg, Rachel Moseley, Kaiping Peng, Jie Sui

**Affiliations:** 1 Department of Psychology, Tsinghua University, Beijing, China; 2 Faculty of Health and Wellness, City University of Macau, Macau, China; 3 School of Psychology, University of Aberdeen, Aberdeen, Scotland, UK; 4 Department of Psychology, Bournemouth University, Poole, UK

**Keywords:** Self, Sex, Selfie editing, Self-prioritization, Perceptual matching task, Life satisfaction, Human behaviour, Population screening

## Abstract

Digital technology has introduced a novel form of self-representation, the selfie. This study investigated the psychological effects of selfie editing and its relationship with well-being by measuring editing behaviors, subjective and objective evaluations of selfies, and life satisfaction. The objective assessment employed a speeded perceptual matching task where participants learned to associate selfies and stranger photos with geometric shapes, then judged the correctness of subsequent photo- shape pairings. Results demonstrated that image editing enhanced immediate selfie satisfaction across sexes. Hierarchical drift diffusion modeling revealed preferential processing of both edited and unedited selfies versus stranger photos, suggesting that edited selfies may function as an extended self-identity. Bayesian regression analysis identified significant associations between life satisfaction and individual differences in selfie perception. Women who processed unedited selfies faster, with higher drift rate, yet reported greater satisfaction with edited selfies, exhibited lower life satisfaction. In contrast, women who demonstrated preferentially processed and reported higher satisfaction with edited selfies, had greater life satisfaction. These associations were absent in men. The findings suggest that congruency of subjective self-evaluation and objective processing of selfies might influence psychological well-being, while indicating sex differences in editing behaviors, underlying cognitive processes, and life satisfaction associations.

## Introduction

The self-face, defined as the mental or visual representation of one’s own facial features—serves as a unique identity symbol that distinguishes individuals from others^1^.With the proliferation of digital technology, selfies (photographic self-portraits typically taken with smartphones or digital devices) have emerged as a novel means for individuals to present their self-face as desired^2,3^. However, the proliferation of digital selfie editing tools challenges this biological mechanism. In the digital era, people use virtual makeup methods like editing their selfies to enhance facial appearance^4,5^. Selfie editing or selfie manipulation refers to beautifying selfies using retouching and beauty software^5^. Editing selfie is a personalized and complex process that typically involves modifying different parts, including internal features like eyes, nose, and mouth, and external features like the shape of face^4,6^. While these modifications may initially enhance satisfaction with one’s appearance, they paradoxically may lead to heightened body dissatisfaction, anxiety, and an increased interest in cosmetic surgery. This raises a critical question: How does altering one’s digital face-image interact with inherent self-recognition processes, and what are the psychological implications of this phenomenon?

These modified ‘self-portraits’ depict how individuals aspire to be perceived in the virtual world. As social animals, people have an innate desire to control how they are perceived by others, this can be achieved through various means of personal grooming or make-up use^7,8^. In the digital era, this is paralleled by use of virtual makeup and selfie editing to enhance facial appearance^4,9^. Empirical evidence on the psychological effects of selfie editing is mixed. While some studies suggest that editing selfies can temporarily boost confidence and social approval, others associate frequent editing with reduced satisfaction with appearance and increased body surveillance. For example, extensive selfie editing has been linked to distorted self-perception and lower body satisfaction^6^. A longitudinal study revealed that even after several months, editing behavior was associated with increased willingness to undergo plastic surgery^10^. While these studies suggest that selfie editing could contribute to lower appearance satisfaction, not all findings support this hypothesis. For example, a study found that editing selfies by filters had no impact on one’s satisfaction with their appearance^11^. However, other researchers reported that people had a more positive evaluation of edited selfies compared to unedited ones^12^, and especially that people tended to overestimate the size of their eyes when editing and representing their facial features when external features of the face are visible^13^.

These disparate findings hint at a critical gap in understanding: current research, relying heavily on explicit self-reports, ignores the subconscious processing of edited self-images and its potential long-term psychological outcomes. In this aspect that the selfie editing changes one’s facial features in selfie images, would the edited selfie still be considered as a part of oneself or an alienated entity? To some extent, does the edited selfie lose some of its ‘self’ attributes? Would this edit alter levels of self-processing, such as higher-level processing in self-report judgements relating to the self, and lower-level self-prioritization effects in cognitive tasks? Consequently, given the relevance of how we represent our self concept to mood and well-being^6,11,14^, would selfie editing affect one’s well-being such as life satisfaction? These questions were explored in the present study.

If selfies are an essential component of self-representation in the digital era, we would expect comparable performance to edited and unedited selfies, coupled with the general prioritization of self-face over strangers, as is typically observed in face recognition regardless of stimulus and task types^15–17^. This is in line with the view that having a stable self-representation is beneficial because it enables individuals to adapt flexibly to different environments and prioritize the processing of information that directly relates to their survival^18–20^, although changes in self-representation have been observed in neurodivergent individuals^21^ and people with depression or anxiety^22,23^. On the other hand, the self-objectification theory proposes that people typically view themselves from their own perspective (first-person), but social and cultural factors can also influence how they perceive themselves from the perspective of others (third-person)^24–26^. The act of editing selfies may relate to the view of self-objectification^27–30^. When taking and editing a selfie, one may play two roles - the producer and the object of the photo^30^. The selfie-taker turns themselves into an object to be observed and evaluated, and imagines how their images will be look like from the perspective of others^14^.

In addition, given that men and women show different patterns of engagement with selfie editing on social media, and are differentially affected by self-objectification, the present study also tested whether effects of selfie editing on face processing might vary as a function of biological sex (also termed ‘sex’ assigned at birth; henceforth ‘sex’). Research has shown that women, compared to men, are more prone to editing their selfies and they are more likely to be exposed to idealized body images on social media and internalize these images^31^. This is consistent with their propensity to be more susceptible to peer pressure and self-objectification^32,33^. A recent study found that exposure to images of slender women on social media increased women’s feelings of body dissatisfaction and the desire to be thin^34^, a pattern less evident in men^35^. As such, it is unclear to what extent sex moderates the relationship between psychological processes of selfie editing and psychological well-being.

In sum, the present study investigated selfie editing behaviors in men and women and the relationship between this digital form of self-representation and psychological well-being, particularly life satisfaction. We examined the data in relation to the following questions:

a) Do men and women differ in selfie editing behaviors and related face satisfaction?

b) How does selfie editing affect face processing? Are there different processes underlying edited and unedited self-face photos compared to photos of strangers?

c) If a selfie represents a digital signature of the self on social media for both men and women, what is the relationship between selfie processing and life satisfaction?

To address these questions, we measured selfie editing behaviors, related satisfaction for edited and unedited selfies, and scores in the Satisfaction with Life Scale^36^. Following selfie editing and satisfaction evaluation of selfies, participants carried out a speeded shape-selfie matching task in which face images (an edited selfie, an unedited selfie, a photo of a stranger) were presented with geometric shapes and they had to judge whether the face-shape pairing matched as originally assigned during the instruction phase^37^. Data analysis employed linear mixed models and Bayesian hierarchical regressions to account for individual variations in selfie editing behaviors. In addition, we utilized Hierarchical Drift Diffusion Model (HDDM) analysis, building on previous studies that have revealed the cognitive processes underpinning self-prioritization, especially in the drift rate, through the application of this computational approach^38–40^.

## Methods

### Participants

One hundred Chinese volunteers (46 women, 54 men. Sex, a key variable in this investigation, was operationalized by asking participants their ‘biological sex’ (choices ‘Men’, ‘Women’ and ‘Other’). While sex is typically assigned at birth in relation to said biological characteristics^41^, it is separate from gender, which is typically understood to describe an individual’s identification with norms and characteristics associated with societally constructed gender roles like ‘man’ and ‘woman’. Though the majority of individuals express gender identities congruent with their sex assigned at birth, some do not. Although we operationalized only sex assigned at birth, gender influences are important in relation to selfie behavior and we highlight their importance for future research, see Discussion; mean age = 22.33 years, *SD* = 2.64, range: 18 to 32 years) participated in the study.

The sample size was determined by a prior power analysis to detect a medium effect size (*r* = 0.30 − 0.45) in the correlation between implicit cognitive measures and self-report measures. Based on previous studies^42^, the analysis indicated that 67 participants were needed to achieve a power of 0.80 at a significance level of α = 0.05 using a two-tailed test. Therefore, a sample size of 100 participants was planned to allow for potential data exclusion. Twelve participants were excluded from the analysis due to their average performance being below the chance level (50%).

The final analysis included 88 participants (42 women, 46 men). All participants were right-handed and had normal or corrected visual acuity. Among them, 30% were graduate students, while the remaining 70% were undergraduates, all of whom were recruited from an internal university forum at Tsinghua University. Informed consent was obtained from all participants prior to participation in the study. The study was approved by the Ethics Committee of the Department of Psychology, Tsinghua University (Ethics Review No. 16, 2019). All methods were implemented in accordance with the Declaration of Helsinki.

### Procedure and materials

**Fig. 1:**
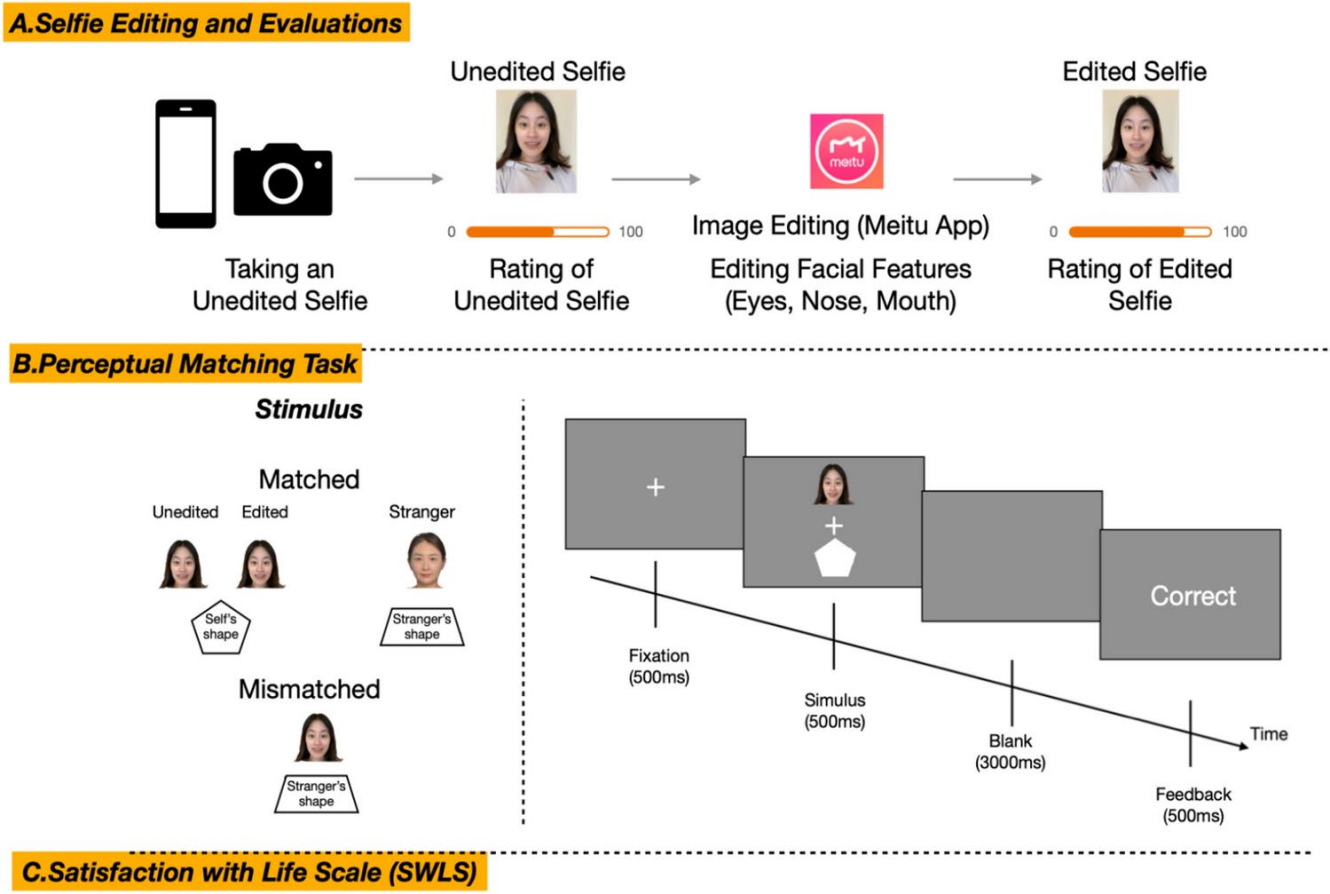
Experimental procedures and tasks. (**A**) Selfie editing and evaluation, (**B**) illustration of a trial procedure in the matching task, with each trial depending on face (Unedited Selfie, Edited Selfie, Stranger’s Selfie), (**C**) Satisfaction with Life Scale (SWLS).

The participants were individually tested in a dedicated laboratory on the campus of Tsinghua University. As illustrated in Fig. 1, first, each participant completed a selfie editing task. Subsequently, they underwent two measurements to evaluate the impact of selfie editing effects on psychological levels. The first measurement assessed self-reported facial satisfaction with both unedited and edited selfies, while the second measured their implicit processing using the perceptual matching task. Finally, they completed the Satisfaction with Life Scale^36^.

#### The selfie editing task and facial satisfaction evaluations

Upon entering the laboratory, participants were instructed to take a selfie, which would serve as an experimental stimulus for the study. To enhance the realism of the selfie-taking experience, participants were informed that their selfies would be printed as Polaroid photos, which they could take home after the experiment. Participants were also encouraged to imagine sharing their selfies on social media. To ensure consistency in photo quality, all selfies were taken using iPhone X in a controlled studio environment with standardized lighting and background conditions. Following the selfie-taking task, participants rated their satisfaction with the unedited selfie using a Visual Analogue Scale (VAS)^6^. The VAS consisted of a horizontal line with vertical markers at each end, representing ‘very dissatisfied’ (0) and ‘very satisfied’ (100), as shown in Fig. 1A.

After evaluating their unedited selfies, participants received a mobile phone (iPhone X) equipped with the standardized selfie editing app (Meitu Xiu Xiu, also known as MeituPic) and were instructed to edit their selfies, focusing specifically on enhancing internal facial features (i.e., the eyes, nose, and mouth) to ensure consistency in the editing process across participants. By limiting the areas subject to modification, we were able to more effectively isolate the effects of editing on facial satisfaction and processing. Participants were encouraged to enhance the visual appeal of their selfies. After editing, participants rated their satisfaction with the edited selfies on a VAS. The editing degree (size) was calculated as the percentage change in pixel-based distances between pre-defined facial landmarks before and after editing.

#### Perceptual matching task

To assess participants’ objective perception of their selfies, we adapted a Shape-selfie perceptual matching task from previous research^37^. This task involved presenting two types of stimuli simultaneously: photos and graphics (See Figure 1B). The photo stimuli included participants’ own selfies (both unedited and edited), as well as selfies of strangers of the same sex. Each participant viewed only one unedited selfie of a stranger, which was randomly selected from a pre-prepared photo library of unedited selfies, ensuring sex consistency with the participant. The stranger’s photo varied across participants. The graphic stimuli consisted of two randomly assigned geometric shapes, such as pentagon and trapezoid. Participants were instructed to learn two shape/photo pairings (e.g., pentagon/selfie, trapezoid /stranger in our example), and were then asked to judge whether each pairing matched (e.g., match: pentagon/selfie; mismatch: pentagon/stranger), as shown in Fig. 1B.

During the matching test, participants completed 12 self-paced practice trials to become familiar with the task. Then, another 12 practice trials were presented at the actual speed with a central fixed cross displayed for 500ms, followed by a shape-photo pair presented for 500ms in the center of the screen, and then a blank screen for 3000ms, during which participants were required to respond quickly and accurately by pressing one of two response buttons (counterbalanced across participants). After each trial, feedback was displayed to participants for 500ms (‘correct’, ‘incorrect’, or ‘too slow’ if no response was made within the response time window). After completing the practice rounds, participants completed eight experimental blocks consisting of 40 trials each, for a total of 320 trials, with 160 trials in each match and mismatch condition (self-photo and stranger-photo were counterbalanced). Participants received feedback on their overall accuracy at the end of each block.

#### Subjective life satisfaction

To evaluate participants’ general well-being, the Satisfaction With Life Scale (SWLS) was administered (7-point Likert scale, 1 = strongly disagree; 7 = strongly agree), with higher scores indicating greater life satisfaction. The scale had an overall internal consistency of a Cronbach’s α of 0.814.

### Data analyses

#### Sex differences in selfie editing

To examine the influence of sex on selfie editing behavior, particularly the extent of editing, we constructed a model that included fixed effects for editing degree, sex, and their interaction, with selfie editing time as a co-variate. To account for individual variability, a random intercept was incorporated for each participant (Editing degree ∼ 1 + Sex + Editing time + (1 | Participant)).

#### Impact of selfie editing

##### Subjective face satisfaction from selfie editing

For the editing effect at the subjective level, a linear mixed model was conducted to analyze the selfie editing effect on self-reported face satisfaction and the effect of sex. The model included fixed effects of editing and sex, as well as a random intercept for each participant (Face Satisfaction ∼ 1 + Editing + Sex + Editing: Sex + (1 | Participant)).

##### Objective cognitive processing of selfie editing

To investigate the cognitive mechanisms underlying selfie processing, we employed the Hierarchical Drift-Diffusion Model (HDDM) to analyze participants’ performance in the perceptual matching task, decomposing reaction time and accuracy into distinct decision-making parameters. HDDM assumes that individuals accumulate information from a noisy environment until sufficient evidence is obtained to initiate a response, capturing cognitive processes through key parameters: drift rate (*v*), non-decision time (*t*0), starting point (*z*), and decision threshold (*a*)^43,44^. We constructed five HDDM models that systematically varied key decision parameters (see Table 1).

**Table 1:** HDDM model comparison and DIC values. The "*" in the model number indicates that this model showed the best fit in the experiment. A lower DIC value indicates better model fit.

Model	Matching	Photos	Sex	DIC
1	*z*	*v*	*v*	6790
2	*z,v*	*v*	*v*	5447
3	*z,v*	*z,v*	*z,v*	3545
4	*v*	*z,v*	*z,v*	5212
5*	*a,v,t,z*	*a,v,t,z*	*a,v,t,z*	-1379

#### Linking selfie editing effects to life satisfaction

To further investigate the effect of selfie editing on well-being, we conducted Bayesian hierarchical linear regression analysis to assess how different levels of selfie editing effects predicted life satisfaction. Our sample displayed a wide range of total SWLS scores, from 7 to 32 (*M* = 19.28, *SD* = 5.8), suitable for regression analysis due to the absence of floor or ceiling effects. We separated the editing effects into subjective and objective components. Subjectively, this was defined as the difference in subjective satisfaction between edited and unedited selfies. Objectively, we measured this by the difference in drift rate (*v*), an index for self-other discrimination occurs during the stage of information accumulation^38–40,42^.

Three Bayesian hierarchical regression models were constructed in Jamovi 2.6.2.0 to predict life satisfaction. Models were sequentially compared using nested Bayes Factor analysis: Model 1 (main effects of sex, face satisfaction, and drift rate) was tested against a null model with only an intercept; Model 2, which added all two-way interactions, was compared to Model 1; and Model 3, including the three-way interaction, was evaluated against Model 2 (See Table 2).

**Table 2:** Results of Bayesian hierarchical linear regression models predicting life satisfaction. Dependent Variable = subjective life satisfaction. "*CI LL*" and "*CI UL*" stands for Confidence Interval Lower Limit and Confidence Interval Upper Limit, Drift rate denotes difference between edited selfie and unedited selfie in drift rate. Face satisfaction refers to difference between edited selfie and unedited selfie in face satisfaction. Sex is coded as 1 for male and -1 for female. Interactions are represented using the ‘ × ’ symbol. ∗ *p* < 0.05, ∗  ∗ *p* < 0.01, ∗  ∗  ∗ *p* < 0.001.

Model	Coefficient	Mean	SD	*BF*inclusion	*CI LL*	*CI UL*	*R*^2^	*BF*_10_
1	Intercept	19.284	0.624	1	18.049	20.41	0.018	0.113
	Sex	0.712	0.639	0.706	-0.289	1.598		
	Face Satisfaction	0.252	0.649	0.379	-0.769	0.89		
	Drift Rate	-9.737	21.641	0.417	-35.366	20.99		
2	Intercept	19.284	0.601	1	17.979	20.371	0.123	0.803
	Sex	0.267	0.633	0.89	-0.623	1.714		
	Face Satisfaction	-0.619	0.713	0.473	-1.417	1.162		
	Drift Rate	-19.79	21.116	0.769	-50.854	22.607		
	Drift rate × Sex	34.235	21.137	1.635	-0.367	68.442		
	Drift rate × Face Satisfaction	55.082	26.289	1.201	0	79.982		
	Face Satisfaction × Sex	1.102	0.681	0.822	-0.018	1.73		
3	Intercept	19.284	0.589	1	18.113	20.455	0.168	3.023
	Sex	0.521	0.632	1.413	-0.736	1.778		
	Face Satisfaction	-1.04	0.728	0.605	-2.488	0.409		
	Drift Rate	-12.607	20.978	1.305	-54.355	29.141		
	Drift rate × Sex	41.151	20.978	2.547	-5.485	98.373		
	Drift rate × Face Satisfaction	46.444	26.094	2.039	-0.597	82.898		
	Face Satisfaction × Sex	1.709	0.728	2.052	0.26	3.157		
	Drift rate × Face Satisfaction × Sex	-54.374	26.094	6.59	-106.303	-2.444		

We further applied the Johnson–Neyman technique to investigate interaction effects by pinpointing the range of drift rate where the interaction between subjective and objective selfie editing effects significantly influenced life satisfaction^45^.

## Results

### Sex differences in selfie editing

**Fig. 2:**
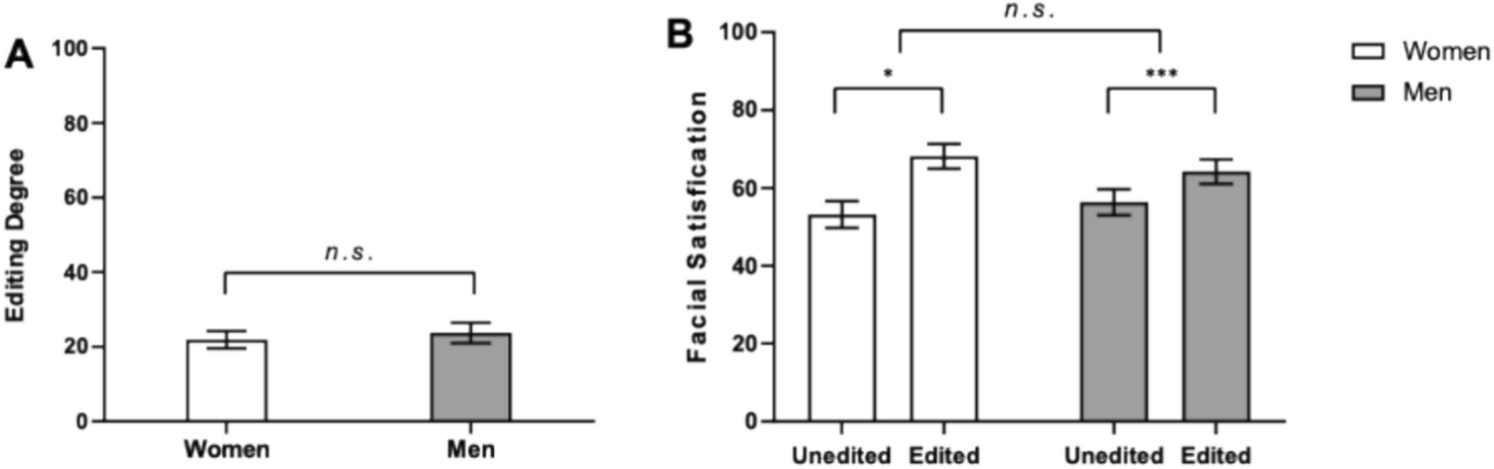
(**A**) Effects of sex on selfie editing and (**B**)face satisfaction. *n.s.* = non-significant difference; *p < 0.05; *** *p* < 0.001.

The results of the analysis examining the effect of sex on selfie editing degree revealed no significant difference between women and men in the extent of selfie editing, sex (*F* (1, 83) = 0.09, *p* = .77), nor in editing time (*F* (1, 83) = 0.07, *p* = .79). As shown in Fig. 2A, these findings indicated that both men and women engaged in a certain degree of selfie editing, with no significant differences between sexes at the behavior level.

### Impact of selfie editing

#### Subjective satisfaction from selfie editing

The analysis demonstrated a significant effect of selfie editing on subjective face satisfaction, β = 11.40, *SE* = 2.01, *t*(86)= 5.67, *p* < .001. Participants reported higher satisfaction with their edited selfies (*M* = 66.10, *SD* = 20.90) compared to their unedited selfies (*M* = 54.80, *SD* = 22.40), suggesting that selfie editing enhances individuals’ subjective evaluations of their own facial appearance (see Fig. 2B).

Furthermore, neither the main effect of sex (β = −0.37, *SE* = 4.19, *t*(86) = −0.09, *p* = .93) nor the interaction between selfie editing and sex (β = −7.06, *SE* = 4.02,*t*(86) = −1.76, *p* = .08) was significant. These findings indicate that the positive effect of selfie editing on face satisfaction was consistent across sexes, suggesting that both men and women derive comparable increases in satisfaction from editing their selfies.

#### Objective cognitive processing of selfie editing

The mathematical modeling of implicit face processing revealed that the full model, Model 5, provided the best fit, as indicated by the lowest DIC value (-1379) (see Table 1). This model incorporated four key decision parameters: decision threshold (*a*), drift rate (*v*), non-decision time (*t*), and starting point (*z*), suggesting that a comprehensive approach is necessary to capture the cognitive mechanisms underlying face-related decision making.

**Fig. 3:**
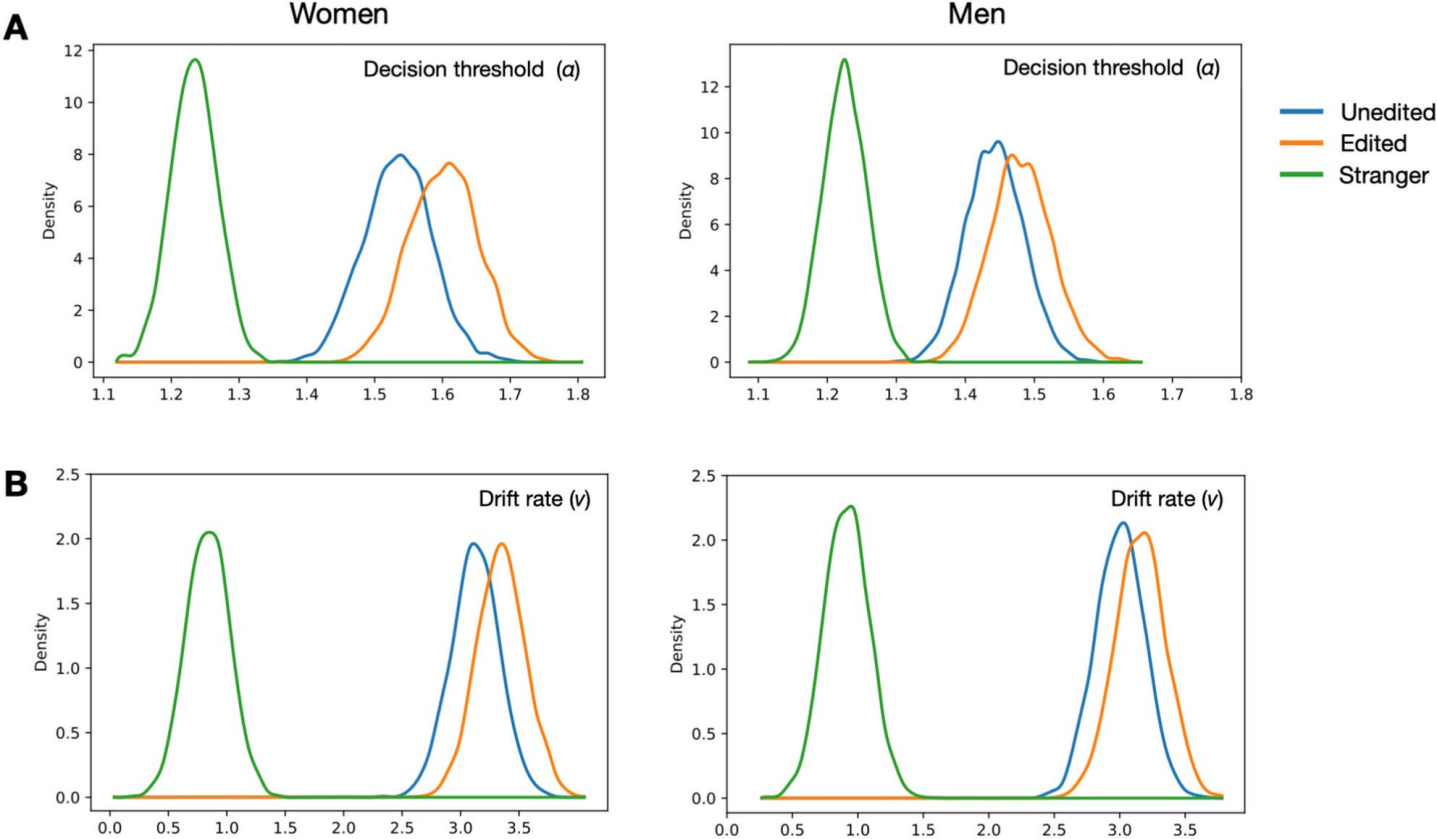
Posterior distributions decision threshold (**A**) and drift rate (**B**)for different photos (unedited, edited, stranger) and sex. Both edited and unedited selfies show larger α and *v* compared to strangers’ photos (*p*Bayes = 1), indicating self-prioritization effect (SPE) in selfies.

The analysis of decision threshold (*a*) provided strong evidence for self-prioritization effects (SPE) in both women and men. Specifically, participants exhibited higher decision thresholds when responding to both unedited and edited selfies compared to strangers’ selfies, indicating greater caution and more conservative decision-making when processing self-related images (*pBayes* [Women Unedited > Stranger] = 1, *pBayes* [Women Edited > Stranger] = 1; *pBayes* [Men Unedited > Stranger] = 1, *pBayes* [Men Edited > Stranger] = 1). However, there was insufficient evidence to suggest a difference in decision thresholds between unedited and edited selfies within each sex (*pBayes* [Women Edited > Women Unedited] = 0.83; *pBayes* [Men Edited > Men Unedited] = 0.75) (see Fig. 3B).

Results revealed that women had a larger drift rate (*v*) for both unedited and edited selfies compared to strangers’ selfies (*pBayes* [Women Unedited > Stranger] = 1, *pBayes* [Women edited > Stranger] = 1](see Fig. 3A). Likewise, men showed a similar pattern of results (*pBayes* [Men Unedited > Stranger] = 1, *pBayes* [Men edited > Stranger] = 1]. However, the analyses showed insufficient evidence for differences between unedited selfie and selfie (*pBayes* [Women Edited > Women Unedited] = 0.78; *pBayes* [Men Edited > Men Unedited] = 0.71).

For non-decisional time (*t*), the analyses demonstrated strong evidence that the non-decisional time was longer on stranger’s selfie for both women (*pBayes* [Women Unedited > Stranger] = 1, *pBayes* [Women Edited > Stranger] = 1]) and men (*pBayes* [Men Unedited > Stranger] = 1, *pBayes* [Men Edited > Stranger] = 1]. No other effect was found on the remaining conditions for non-decisional time (*pBayes* < 0.5).

No significant differences in the starting values (*z*) analyses were observed in comparisons between non-biased (*z* = 0.50) and selfie conditions (*pBayes* < 0.5 = 1).

The results indicated that while selfie editing modifies specific visual features, it did not substantially change individuals’ perception of their own facial identity or self-relevance. Selfies, even after editing, are still processed as integral components of the self.

### Linking selfie editing effects to life satisfaction

The results of the Bayesian hierarchical linear regression analysis examining the relationship between life satisfaction and various predictor variables were reported. Three different models were tested; see Table 2.

Model 1 has an *R*^2^ of 0.018 and a *BF*_10_ of 0.113, indicating that the model explains only a very small portion of the variance in life satisfaction. Model 2 has an *R*^2^ of 0.123 and a *BF*_10_ of 0.803. This model adds the interactions between Drift Rate × Face Satisfaction Difference and Drift Rate × Sex to Model 1. After incorporating these interactions, the Bayesian factor (*BF*_10_) for Model 2 increases to 7.137, meaning it receives 7.137 times more support than Model 1, indicating that the inclusion of these interaction terms significantly improved the model’s fit. Model 3 has an *R*^2^ of 0.168 and a *BF*_10_ of 3.023, indicating strong support for the model. Model 3 provides the best fit, particularly after including the three-way interaction. Compared to Model 2, Model 3 fits the data better, with a *BF*10 of 3.764, suggesting that the inclusion of the three-way interaction significantly enhanced the model’s fit, explaining 16.8% of the variance. The coefficient for the three-way interaction (Drift Rate × Face Satisfaction × Sex) is -54.374, with a *BF*inclusion of 6.59 and a 95% credible interval of [-106.303, -2.444], providing strong evidence for the impact of this interaction on life satisfaction.

**Fig. 4:**
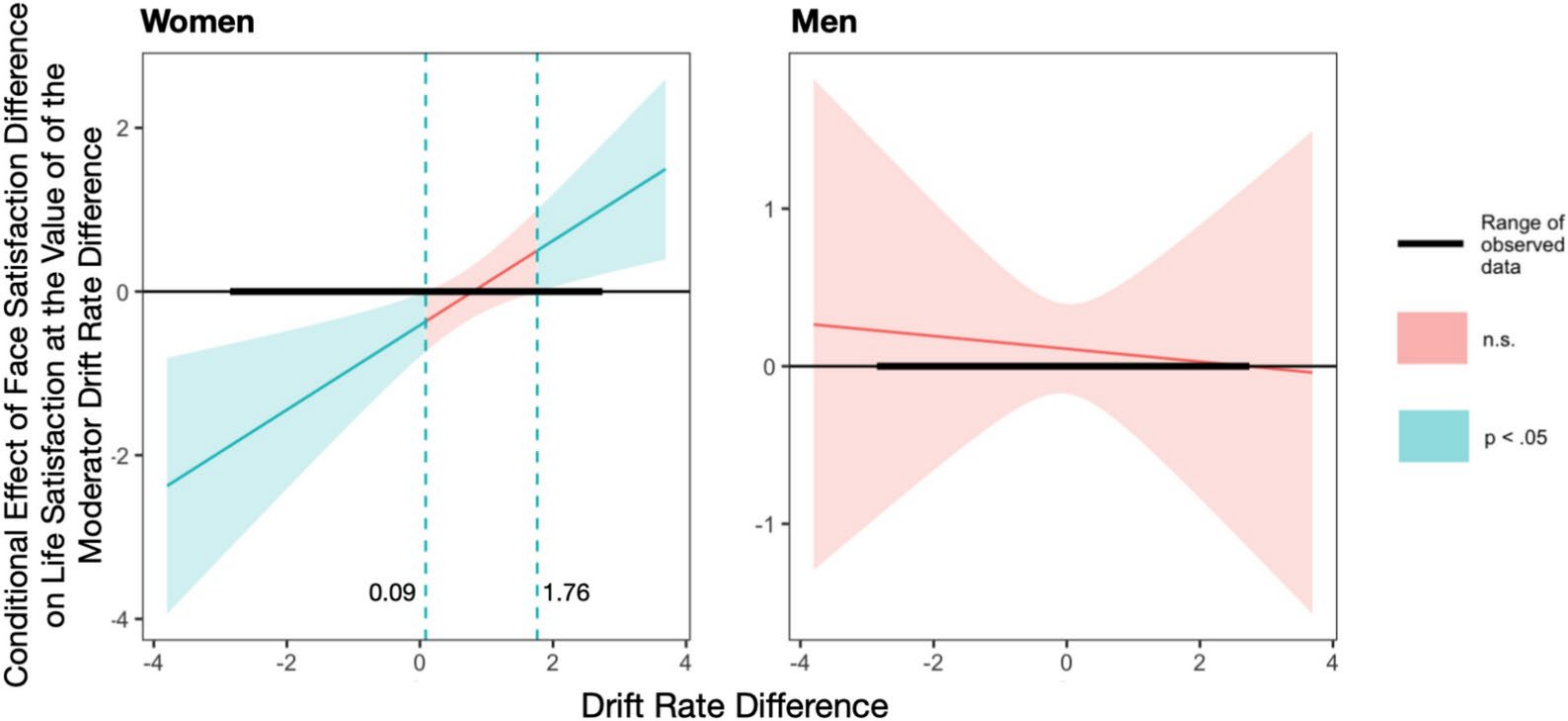
Effect of face satisfaction difference, drift date difference, and sex on life Satisfaction. Johnson-Neyman plot shows how the face satisfaction difference influences life satisfaction, depending on different levels of the moderator, drift rate difference. The Y-axis represents the slope of the face satisfaction difference, indicating the strength and direction of its relationship with life satisfaction at varying levels of the difference in drift rate. Values below 0 on the horizontal axis represent a negative effect, while values above 0 indicate a positive effect.

Further analysis using the Johnson-Neyman analysis was conducted to investigate the moderating effect of sex on the relationship between Drift Rate Difference and Face Satisfaction Difference influencing life satisfaction. For women the Johnson-Neyman interval was established at [0.09, 1.76], see Fig. 4. When the selfie editing effects in drift rate was below 0.09, women showed a negative significant relationship between face satisfaction with edited selfies and life satisfaction, indicating that although they were more satisfied with the edited selfies, their faster processing of unedited selfies was associated with lower life satisfaction. In contrast, when the drift rate difference exceeded 1.76, the relationship between selfie editing effects in face satisfaction and life satisfaction became positively significant, suggesting that when implicit and explicit processing aligned (i.e., both favorable toward the edited selfies), life satisfaction increased. For men, the simple slopes analysis showed no significant effects of selfie editing on life satisfaction at any level of drift rate difference (*p* > .05), indicating that the moderating effect of sex was only observed in women.

In summary, life satisfaction in women was influenced by the consistency between implicit and explicit processing of selfies: when there was a conflict (i.e., higher satisfaction with edited selfies but faster processing of unedited selfies), life satisfaction was lower; when there was alignment (i.e., both implicit and explicit processing favor the edited selfies), life satisfaction was higher. In contrast, such patterns were not observed in men, suggesting potential sex differences in self-presentation strategies.

## Discussion

We examined selfie editing behaviors and the association between this digital form of self-representation and life satisfaction. Our investigation revealed three key findings. First, the results showed that selfie editing increased face satisfaction with selfie images in both men and women, suggesting a general positive effect of digital self-modification on immediate self-perception. Second, hierarchical drift diffusion model analysis revealed no differences in responses to edited and unedited selfies, though there were robust self-prioritization effects for edited and unedited selfies over photos of strangers. The results indicated that even though selfie editing altered the facial features of face images, people still perceived them as a part of themselves as extended self-identity markers in social media contexts. Third, Bayesian regression analysis revealed sex-specific associations between life satisfaction and individual differences in selfie perception. Women who exhibited faster processing (higher drift rate) of unedited versus edited selfies, while reporting greater satisfaction with edited images, showed lower life satisfaction. In contrast, women who demonstrated preferential processing of edited selfies coupled with higher satisfaction for these images reported greater life satisfaction. Notably, these associations were absent in men. Together, these results are suggestive of sex differences in selfie editing and the associations between cognitive processes of selfies and life satisfaction.

For subjective facial satisfaction, we found that editing selfies significantly increased participants’ satisfaction with their own faces. This result is consistent with previous findings and this was true for both men and women^6^. However, it is important to recognize that initial increases in satisfaction might be short-lived: one study reported that although selfie editing enhanced short-term satisfaction with appearance, this leads to long-term negative effects due to heightened appearance expectations^10^. Thus, while our results align with previous research in suggesting that selfie editing can boost immediate satisfaction with one’s face, the potential for long-term dissatisfaction due to heightened appearance expectations is a significant concern. Future research should further explore this dynamic.

When edited and unedited selfies were presented in the matching task, we observed a robust self-prioritization effect over strangers, but there was no difference in responses to edited and unedited selfie images. The maintenance of self-prioritization effects for both edited and unedited selfies provide compelling evidence for the theory of online self-extension^46^. HDDM analyses further revealed that this advantage was stable across different stages of decision-making, consistent with previous studies^47,48^. The lack of difference between edited and unedited selfies supports the view that edited selfies are a facet of self-representation^9^. This is also in line with research on self recognition, which demonstrates that processing one’s own faces has a consistent advantage over processing the faces of others, regardless of stimulus type and task-relevance^15,49^.Despite digital modifications, individuals process edited selfies not as ‘foreign’ entities but as extensions of self-identity, demonstrating remarkable stability in self-recognition mechanisms. This evolutionary-rooted stability suggests that our fundamental self- recognition systems exhibit some resistance to rapid changes while maintaining sufficient flexibility to accommodate digital self-modifications.

However, individual differences in processing between edited and unedited selfies revealed psychological vulnerabilities, particularly among women. When women processed unedited selfies more efficiently than edited ones, indicated by faster drift rates, while expressing preference for edited images, they tended to experience decreased life satisfaction. This cognitive- behavioral discrepancy may serve as an objective indicator of internal conflict. From the self-objectification theory, women are more likely than men to be exposed to idealized body images on social media and internalize these image^50^. It is possible that societal pressures and expectations regarding beauty standards could play a role, where extensive editing might be perceived as inauthentic or trying too hard, which could negatively impact self-perception and, consequently, life satisfaction^14,30^. Notably, when women’s processing for edited selfies aligned with their subjective satisfaction, this cognitive behavioral congruency reflected successful integration of digital modifications into extended self-boundaries, associated with higher life satisfaction. This cognitive behavioral integration may indicate an individual’s ability to flexibly integrate their selves across the real and virtual worlds, and this psychological adaptation is associated with higher psychological well-being^51,52^. Conversely, men showed no significant associations between processing selfies and life satisfaction, likely reflecting reduced society appearance pressures and less frequent appearance-based evaluation^31,35^. We report preliminary exploratory findings in the supplementary information.

There are some implications for our findings. First, the sample for this study was taken from an East Asian cultural background. According to the objectification theory of self, individuals are socialized by the objective experiences they encounter on a daily basis and internalize external aesthetic standards^24^. For example, in some East Asian societies where historical and media-driven beauty standards prioritize slimmer body types and lighter skin tones, frequent engagement in appearance-related social comparisons may exacerbate body dissatisfaction^53^.Therefore, the current study’s results may be ungeneralizable outside of East Asian and future research could consider comparing samples from different cultural backgrounds. Relatedly, while we focused on sex assigned at birth, our design did not model or operationalize gender as a separate construct. Sex and gender have been binarized and conflated in prior investigations of selfie behavior^29,30^, but their effects should be delineated and conceptualized more expansively in future work, particularly given that selfie editing can also be a means through which gender can be expressed^54^.Third, the small sample size limited statistical power to detect certain interactions. Future studies with larger, diverse samples are needed to confirm these results.

In summary, the present study for the first time measured the subjective and objective effects of self-editing on face processing for men and women and its relationship with life satisfaction. These findings revealed that selfie editing enhances immediate facial satisfaction across men and women, and there were similar processes underpinning edited and unedited self-face processing in both men and women, which leads to an advantage effect over processing face images of strangers. However, the relationship between selfie editing and psychological well-being appears to operate through sex-specific processes, with women showing particular sensitivity to processing discrepancies between edited versus unedited self-images.

## Supplementary Information

Supplementary Information 1.

Supplementary Information 2.

## Data Availability

The datasets generated and analyzed during the current study are available in the Open Science Framework (OSF) repository, https://osf.io/ea9kd/.
