# Supplementary material for: Digital selfie editing shows sex specific associations between processing biases and life satisfaction
Source: Sci Rep. 2025 Apr 24;15:14235. doi: 10.1038/s41598-025-99056-y (PMC12022245; doi:10.1038/s41598-025-99056-y)
Supplement: Supplementary file 1 — Supplementary Information 1. [file 41598_2025_99056_MOESM1_ESM.pdf]

Additional information

Supplemental materials

Results

To further examine the effect of different facial feature editing behaviors in men and women on life satisfaction, we conducted another regression model. First, we added the degree of editing for different internal features (eyes, nose, and mouth), differences in the drift rate, and sex to Model 1. Model 2 further added two-way interaction effects between variables and the interaction effects on the difference in drift rate and sex and editing of different internal features (see Supplementary Table 1).

The model results showed that the results of Model 1 were not significant, $F(5, 82) = 1.72$, $p = .22$, adjusted $R^2 = .04$; Model 2 predicted life satisfaction significantly, $F(15, 72) = 2.36$, $p = .01$, adjusted $R^2 = .33$, in which three predictors significantly predicted life satisfaction: the interaction between sex and drift rate difference ($b = 0.25$, $p < .05$), the interactions between sex and drift rate difference and eyes editing ($b = 0.36$, $p < .05$), and the interactions between sex and drift rate difference and mouth editing ($b = 0.25$, $p < .05$).

Further analysis of the interaction showed that when the difference in drift rate was above 1SD, women's life satisfaction was higher than men ($b = 0.33$, $t = 2.10$, $p < .05$). However, when differences in drift rates were at average levels ($b = 0.12$, $t = 1.06$, $p = .29$) or below 1SD ($b = 0.10$, $t = 0.64$, $p = .52$), there was no sex effect on life satisfaction.

Regression analyses explored the role of specific facial feature editing behaviors. These models included editing degree (eyes, nose, and mouth), drift rate difference, and sex as predictors. In Model 1, the results were not significant ($F(5, 82) = 1.72$, $p = .22$, adjusted $R^2 = 0.04$). However, Model 2, which added interaction terms, significantly predicted life satisfaction ($F(15, 72) = 2.36$, $p = .01$, adjusted $R^2 = 0.33$). Significant predictors included the interaction between sex and drift rate difference ($b = 0.25$, $p < .05$), sex × drift rate difference × eyes editing ($b = 0.36$, $p < .05$), and sex × drift rate difference × mouth editing ($b = 0.25$, $p < .05$).

Eyes editing: For men, higher editing of eyes (+1SD) was associated with a stronger positive relationship between drift rate difference and life satisfaction ($b = 0.61$, $t = 2.83$, $p < .01$), but no significant relationships were observed for women across all editing levels ($p > .05$). See Supplementary Figure 1A.

Nose editing: The Johnson-Neyman interval could not be found; See Supplementary Figure 1B.

Mouth editing: For women, lower levels of mouth editing were associated with a negative relationship between drift rate difference and life satisfaction ($b = 0.77$, $t = 2.96$, $p < .01$). However, no significant effects were observed for men across all editing levels ($p > .05$). See Supplementary Figure 1C.

Discussion

An analysis revealed that sex moderates the relationship between selfie editing, particularly of the mouth, and life satisfaction. Specifically, women who minimally edited their mouths and had a slower information uptake towards unedited selfies experienced higher life satisfaction. This aligns with the notion that editing features such as the mouth can have a substantial impact on how women perceive themselves, possibly due to cultural emphasis on lips as a beauty standard. For instance, lipsticks have been culturally significant and may have influenced women's perceptions of their lips[50]. Moreover, fuller lips in women are suggested to be cues for health and reproductive capabilities[55]. A study about facial cosmetics found that facial cosmetics, especially around the mouth, significantly alter face perception, as evidenced by changes in the N170 event-related potential component[56]. However, this relationship between editing and life satisfaction was not observed for women who edited their mouths more extensively. This could be due to a variety of factors. For instance, more extensive editing might create a larger discrepancy between the edited image and the individual's actual appearance, which could lead to dissatisfaction with one's real appearance. Additionally, extensive editing might reflect an individual's existing dissatisfaction with their appearance, and the edited image might not sufficiently address the underlying concerns or insecurities. It's also possible that societal pressures and expectations regarding beauty standards could play a role, where extensive editing might be perceived as inauthentic or trying too hard, which could negatively impact self-perception and, consequently, life satisfaction.

Conversely, among men who extensively edited their eyes in selfies, there was a positive correlation between the speed of information accumulation towards edited selfies (greater drift rate) and life satisfaction. This was not the case for men who engaged in average or below-average levels of eye editing. This finding aligns with a previous study, which revealed that individuals tend to overestimate the size of their eyes when editing and representing their facial features[13]. This suggests that editing features such as the eyes can be particularly influential in how one perceives faces, which may explain why men who extensively edit their selfie eyes and have a faster information accumulation rate towards the edited selfies experience higher life satisfaction.

Supplementary Table 1. Results of multilevel regression analysis on the effects of selfie editing and sex on life satisfaction. Eye, Nose, and Mouth describe the degree of editing on these features. Drift Rate Difference refers to the difference in information accumulation rates between edited and unedited selfies. Sex is coded as 1 for male and -1 for female. *$p < .05$.

Model	Predictors	B		SE(B)	β	Adj R^2	ΔR^2	F
1	Intercept	-0.01		0.11		0.04		1.72
	Eye	-0.15		0.13	-0.15			
	Nose	-0.13		0.12	-0.13			
	Mouth	-0.09		0.12	-0.09			
	Sex	0.14		0.11	0.14			
	Drift Rate Difference	0.01		0.11	0.01			
2	Intercept	0.02		0.1		0.19	0.15*	2.36*
	Eye	0		0.15	0			
	Nose	-0.2		0.13	-0.2			
	Mouth	-0.24		0.14	-0.24			
	Sex	0.18		0.1	0.18			
	Drift Rate Difference	-0.07		0.11	-0.07			
	Drift Rate Difference × Sex	0.25	*	0.11	0.25			
	Drift Rate Difference × Eyes	0.07		0.17	0.07			
	Sex × Eyes	0.05		0.15	0.05			
	Drift Rate Difference × Mouth	0.18		0.12	0.18			
	Sex × Mouth	0.01		0.14	0.01			
	Drift Rate Difference × Eyes	0.08		0.17	0.08			
	Sex × Nose	-0.01		0.13	-0.01			
	Drift Rate Difference × Sex × Mouth	-0.26	*	0.12	-0.26			
	Drift Rate Difference × Sex × Nose	-0.25		0.19	-0.25			
	Drift Rate Difference × Sex × Eyes	0.36	*	0.17	0.36			

The observed sex differences in the relationship between selfie editing of facial features and life satisfaction can be elucidated through previous studies[56, 57]. One study demonstrated that eyes are central to face perception, while the mouth is more associated with communication[57]. This aligns with findings that men, who often edit their eyes, experience higher life satisfaction, likely due to the eyes' role in face perception. In contrast, women frequently edit their mouths, reflecting societal beauty standards and communication aspects. The other study further supports this by showing that cosmetics, particularly around the mouth, significantly influence face perception[56]. Together, these studies highlight how sex-specific editing preferences, rooted in the fundamental roles of facial features, can differentially impact life satisfaction.

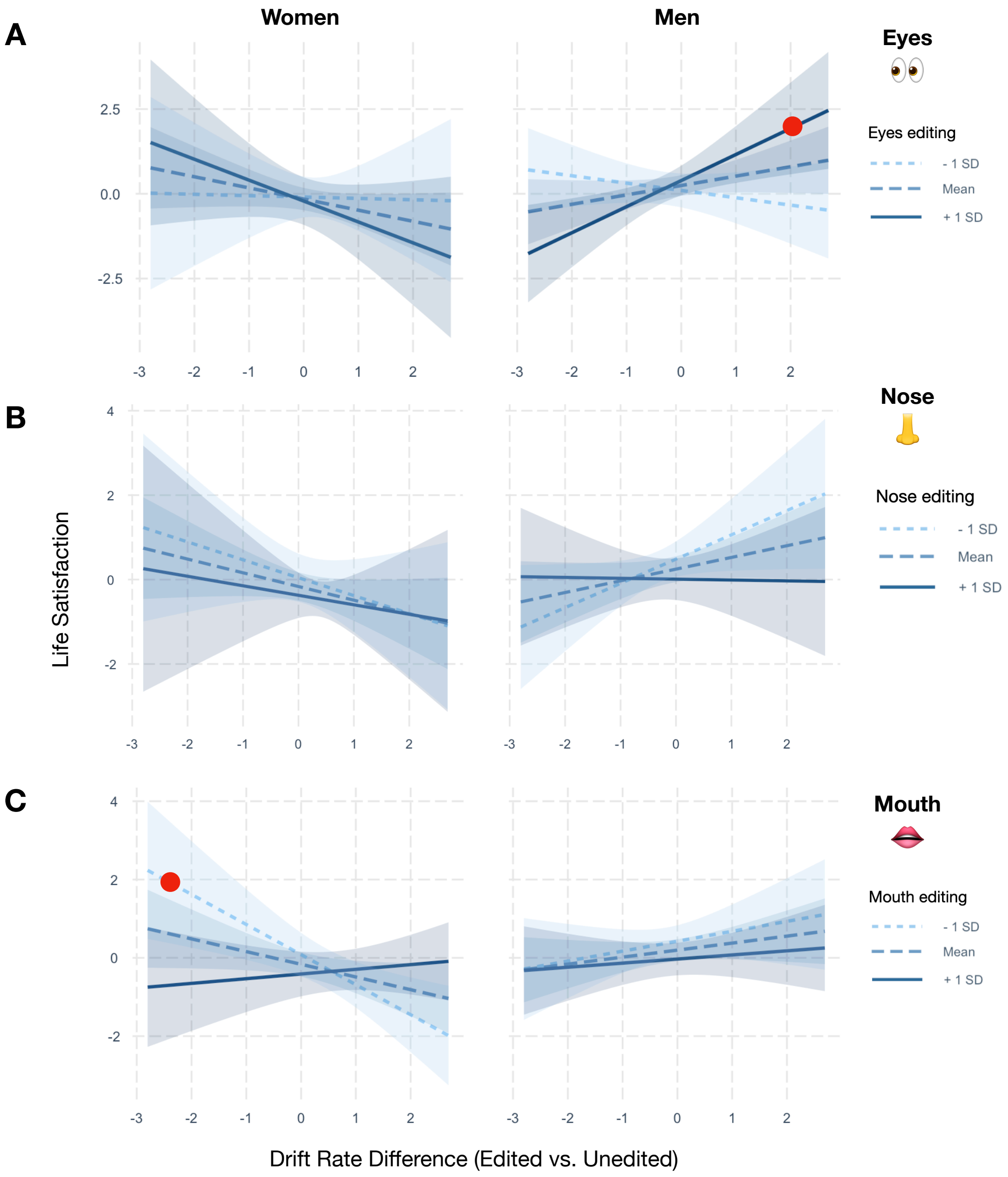

Supplementary Figure 1. Effect of drift rate difference (Edited vs. Unedited) and sex on life satisfaction - eyes editing (A), and nose editing (B) and mouth editing (C). Simple slope test results showing the interaction between facial feature editing (eyes, nose, and mouth) and drift rate difference (Edited vs. Unedited) on life satisfaction for both women and men. The plots depict the relationship between the drift rate difference and life satisfaction at different levels of facial feature editing, with the shaded areas representing standard deviations. Red Dots represent significant conditions for life satisfaction.

References

50. Etcoff, N. *Survival of the Prettiest: The Science of Beauty* (Anchor, 2011). ISBN: 978-0385479424.
51. Hung, K., Lee, N. A., Peng, K. & Sui, J. Profile pictures in the digital world: Self-photographs predict better life satisfaction. *Int. J. Environ. Res. Public Heal.* 18, 6667, DOI: 10.3390/ijerph18126667 (2021).
52. Lin, X., Su, W. & Potenza, M. N. Development of an online and offline integration hypothesis for healthy internet use: Theory and preliminary evidence. *Front. Psychol.* 9, 492, DOI: 10.3389/fpsyg.2018.00492 (2018).
53. Myers, T. A. & Crowther, J. H. Social comparison as a predictor of body dissatisfaction: A meta-analytic review. *J. Abnorm. Psychol.* 118, 683–698, DOI: 10.1037/a0016763 (2009).
54. Goetz, T. G. Self(ie)-recognition: Authenticity, passing, and trans embodied imaginaries. *Stud. Gend. Sex.* 23, 256–278, DOI: 10.1080/15240657.2022.2119980 (2022).
55. Puts, D. A. Beauty and the beast: Mechanisms of sexual selection in humans. *Evol. Hum. Behav.* 31, 157–175, DOI: 10.1016/j.evolhumbehav.2010.02.005 (2010).
56. Tanaka, H. Facial cosmetics exert a greater influence on processing of the mouth relative to the eyes: Evidence from the n170 event-related potential component. *Front. Psychol.* 7, 1359, DOI: 10.3389/fpsyg.2016.01359 (2016).
57. Key, A. P. F., Stone, W. & Williams, S. M. What do infants see in faces? erp evidence of different roles of eyes and mouth for face perception in 9-month-old infants. *Infant Child Dev.* 18, 149–162, DOI: 10.1002/icd.600 (2009).
