# Supplementary material for: Digital selfie editing shows sex specific associations between processing biases and life satisfaction
Source: Sci Rep. 2025 Apr 24;15:14235. doi: 10.1038/s41598-025-99056-y (PMC12022245; doi:10.1038/s41598-025-99056-y)
Supplement: Supplementary file 2 — Supplementary Information 2. [file 41598_2025_99056_MOESM2_ESM.pdf]

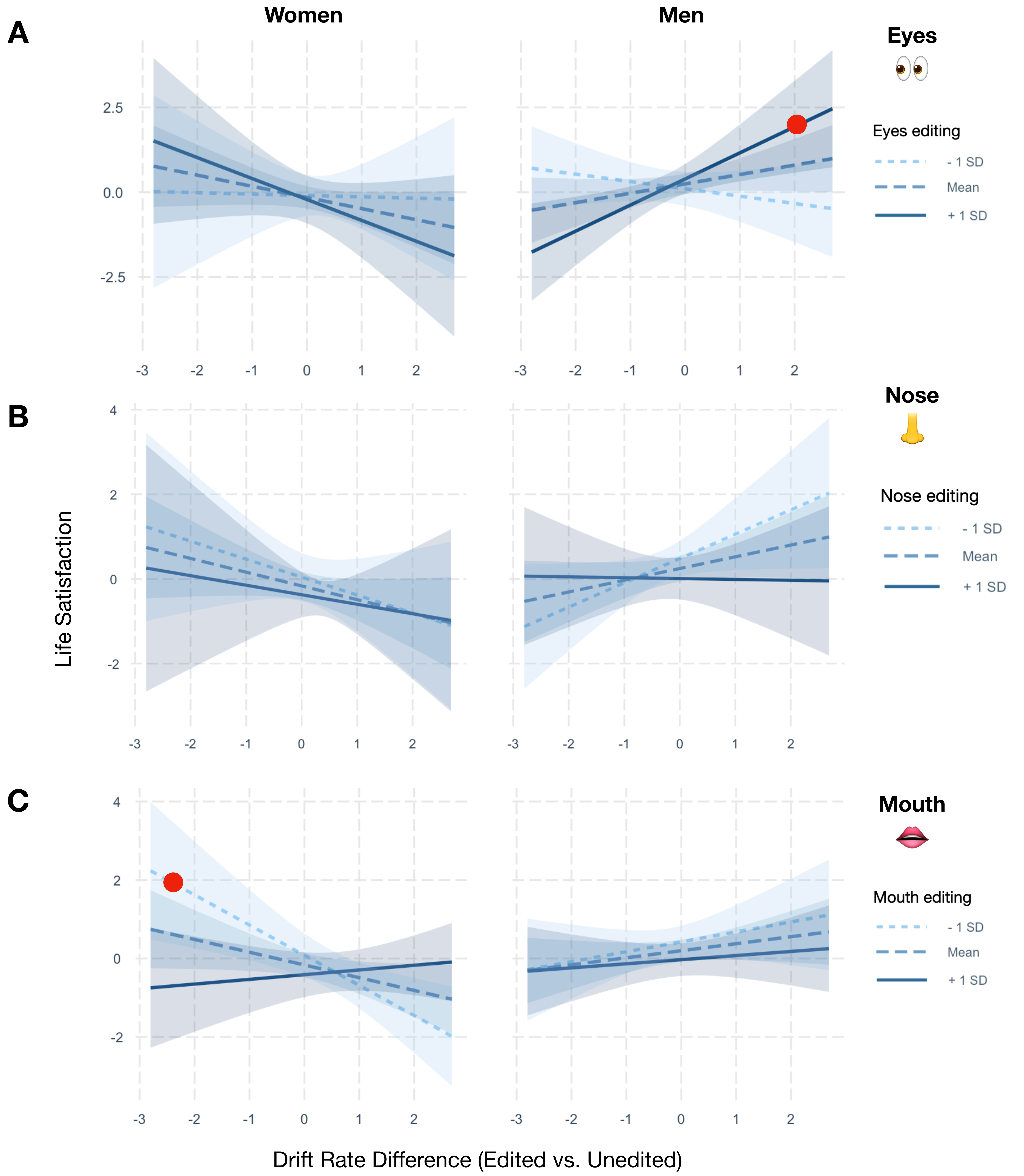

A
B
C
Women
Men
Eyes
Eyes editing
- 1 SD
Mean
+ 1 SD
Nose
Nose editing
- 1 SD
Mean
+ 1 SD
Mouth
Mouth editing
- 1 SD
Mean
+ 1 SD
Life Satisfaction
Drift Rate Difference (Edited vs. Unedited)
2.5
0.0
-2.5
4
2
0
-2
-3
-2
-1
0
1
2
